# Television-Viewing Characteristics of Adults: Correlations to Eating Practices and Overweight and Health Status

**Published:** 2006-03-15

**Authors:** Shanthy A Bowman

**Affiliations:** Agricultural Research Service, Community Nutrition Research Group, Beltsville Human Nutrition Research Center, United States Department of Agriculture

## Abstract

**Introduction:**

The purpose of this study was to examine the associations among television viewing, eating practices, and overweight and health status of a nationally representative sample of adults in the United States.

**Methods:**

Data on adults aged 20 years or older from the U.S. Department of Agriculture's Continuing Survey of Food Intakes by Individuals 1994–1996 were used for the study. Participants' socioeconomic and demographic characteristics, macronutrient intakes, weight status, prevalence of health conditions, television viewing, and overweight status were analyzed. Survey design effects were used in the analyses.

**Results:**

More than 2 hours of television viewing per day was associated with a high mean body mass index and overweight or obesity in both men and women. Other characteristics associated with watching more than 2 hours of television per day were being 50 years of age or older, having a high school education or less, living in a household with income below 131% of the federal poverty level, and not being employed. Adults who watched more than 2 hours of television per day had high intakes of energy and macronutrients and were more likely to be overweight. They also obtained more energy from snacks and supper. A higher percentage of adults with health conditions watched more than 2 hours of television per day compared with adults without health conditions.

**Conclusion:**

Obesity intervention programs, especially those aimed at adults who are retired or not employed, should emphasize reducing time spent viewing television or videos or participating in similar sedentary activities and discourage snacking or eating while watching television.

## Introduction

Currently, 65.7% of U.S. adults are overweight or obese (body mass index [BMI] ≥25 kg/m2^2^), and 30.6% are obese (BMI ≥30 kg/m2^2^) (1). Obesity affects quality of life and increases health care costs (2-8). The adult obesity-attributable medical expenditure in the United States in 2003 was estimated at $75 billion (8).

Television viewing is a popular leisure-time activity (9) and promotes a sedentary lifestyle by infringing on the time available for physical activity. Many researchers have reported a positive association between hours spent watching television and overweight or obesity status (10-19). In a study on 74 women seeking obesity treatment, Gore et al found that snacking while watching television was associated with increased intakes of total calories and calories from fat(20). They did not observe an association between eating meals while watching television and total caloric and fat intakes. Therefore, television viewing may not only facilitate low energy expenditure but also increase energy intake and hence may play an important role in the current obesity epidemic.

The objectives of this study were 1) to assess the relationship between television viewing and weight status of adults from different socioeconomic and demographic groups and 2) to examine whether positive relationships exist between television viewing and total daily energy intakes and daily energy intakes from eating occasions (i.e., meals).

## Methods

The study used a nationally representative sample of noninstitutionalized adults aged 20 years and older from the U.S. Department of Agriculture's (USDA's) Continuing Survey of Food Intakes by Individuals 1994–1996 (CSFII 1994–1996) (21,22). The USDA periodically conducts dietary surveys to monitor the food consumption patterns of households and individuals in households (23). The CSFII sample is a stratified, multistage, area probability sample of U.S. households (24).

CSFII 1994–1996 dietary data were collected on 2 nonconsecutive days, 3 to 10 days apart. The self-reported data were collected through an interviewer-administered, 24-hour dietary-recall method, using a multipass technique to reduce underreporting of foods by respondents (24). Overall, the response rate for the first day of the survey was 80.0%. Adults aged 20 years and older who had complete food intake records and television-viewing information on both survey days were included in the study (N = 9157).

CSFII collects self-reported data on height and weight of survey respondents. Only one question in CSFII 1994–1996 asked respondents how many hours they watched television or a videotape per day. The following choices were available to the respondent: no television watched (or 0 hours), 1 hour or less (coded as 1 hour), integers including 2 through 24 hours, don't know, and not ascertained. Respondents who watched television between 0 and 24 hours on both days of the survey were included in the analyses.

Two-day averages were used for estimating daily hours of television (or videotape) viewed and dietary intakes. The sample was divided into three groups based on the 2-day mean television- and videotape-viewing status: watched less than 1 hour of television per day (n = 1296); watched 1 or 2 hours of television per day (n = 2317); and watched more than 2 hours of television per day (n = 5544). Mean television hours per day were estimated for normal weight, overweight, and obese adults. BMI values were based on self-reported height and weight data. The mean BMI and percentage of overweight and obese men and women in the three television-viewing categories were estimated. Pair-wise mean comparisons within sex categories were made. Other socioeconomic and demographic characteristics and the employment status of survey participants watching more than 2 hours of television and the percentage of overweight adults in the three television-viewing categories were estimated. Pair-wise mean comparisons were made among overweight adults within the three television-viewing categories.

The associations between television viewing and health conditions were examined. The CSFII asked respondents whether a doctor had ever told them that they had health conditions such as diabetes, hypertension, heart disease, or high blood cholesterol. Percentages of adults who watched more than 2 hours of television and percentages of overweight adults in the three television-viewing categories were estimated. Pair-wise mean comparisons were made among overweight or obese adults within each health-condition category. Unadjusted mean macronutrients (i.e., total fat, saturated fat, total carbohydrate, added sugars, and dietary fiber), total energy, energy intakes from different eating occasions, and selected food group intakes of adults in the three television-viewing categories were estimated. Pair-wise mean comparisons were made within the three television-viewing categories.

In the second set of analyses, multiple regression models were used to control for variations in age, sex, race and ethnicity (Hispanic, non-Hispanic white, non-Hispanic black or African American, and other non-Hispanic races such as Asian, Pacific Islander, American Indian, and Alaska Native), annual household income as a percentage of federal poverty level (low income, 0%–130%; medium income, 131%–350%; high income, more than 350%), region (Northeast, Midwest, South, and West), and urbanization (metropolitan statistical area [MSA], including MSA–central city, MSA–suburban, and non-MSA–rural). CSFII included only one question on the exercise status of the respondents. Responses included 1) exercised vigorously daily, 2) exercised 5 to 6 times per week, 3) exercised 2 to 4 times per week, 4) exercised once a week, 5) exercised 1 to 3 times per month, 6) don't know, 7) and not ascertained. In the third step of analyses, exercise status of the respondents was added to the independent variables in the second set of multiple regression models.

The names of eating occasions were as reported by the survey respondents. *Snacks* included beverage breaks. *Regular soft drinks* included all carbonated soft drinks except unsweetened and sugar-free types. *Energy-rich snack-type* foods included cakes, cookies, pastries, pies, sweet rolls, crackers, popcorn, pretzels, corn chips, and fried potatoes, including potato chips. *Grain mixtures* included food mixtures having a grain product as a main ingredient, such as pizza, burritos, tacos, egg rolls, quiche, spaghetti with sauce, rice, and pasta mixtures.

Survey design effects were included in the data analyses using SAS-Callable SUDAAN for Solaris version 9.0.0 July 2004 (Research Triangle Institute, Research Triangle Park, NC). Therefore, all statistics reported in this paper are weighted to reflect the national population. The statistical significances reported are based on pair-wise mean comparisons. For mean comparisons reported in this study, the level of significance α = .05 was selected a priori.

## Results

Overall, 14.7% (95% confidence interval [CI], 13.5%–16.1%) of adults watched less than 1 hour of television per day, 26.4% (95%CI, 24.8%–28.0%) watched 1 to 2 hours of television per day, and 58.9% (95% CI, 56.7%-61.0%) watched more than 2 hours of television per day. Normal-weight adults (BMI = 18.5–24.9) spent significantly less time watching television than overweight or obese adults. The 2-day mean television hours were 2.3 hours (95% CI, 2.2–2.4 hours) for normal-weight adults, 2.6 hours (95% CI, 2.5–2.7 hours) for overweight adults, and 3.0 hours (95% CI, 2.85–3.15 hours) for obese adults. Both men and women who watched more than 2 hours of television per day had a significantly higher mean BMI than those who watched less than 1 hour of television per day (Table 1). In addition, a significantly higher percentage of overweight and obesity was observed among both men and women who watched more than 2 hours of television per day compared with those who watched less than 1 hour.

About half of adults aged 20 to 50 watched more than 2 hours of television per day (Table 2). Also, the percentage of adults who watched more than 2 hours of television in the age groups 20 to 29 years, 30 to 39 years, and 40 to 49 years did not differ significantly  among groups. In contrast, almost three fourths of adults aged 66 years or older watched more than 2 hours of television per day. A strong negative association was seen between level of education and hours of television watched. A low level of education was associated with more television viewing. About two thirds of adults with a high school education or less watched more than 2 hours of television per day; only about half of the adults with 4 or more years of college education watched this amount. More adults from low-income households (0%–130% federal poverty level) than adults from high-income households (more than 350% federal poverty level) watched more than 2 hours of television. African Americans were more likely to watch more than 2 hours of television per day than other racial and ethnic groups. Moreover, more than half of the adults living in the United States, regardless of whether they lived in central cities or suburban or rural areas, watched more than 2 hours of television per day.

Employment status influenced television-watching time. A lower percentage of employed adults than not-employed adults watched more than 2 hours of television (Table 3). The reasons for not being employed included being retired, keeping house, inability to work, looking for work, or going to school. There were sufficient sample sizes in the retired group (n = 2031) and the keeping-house group (n = 673), and their television-viewing time and overweight status were further examined (Table 3). More retired adults than adults who kept house watched more than 2 hours of television per day. Consistently, within all employment status categories, high percentages of adults who watched more than 2 hours of television per day were overweight.

A positive relationship was seen between viewing more than 2 hours of television per day and having a health condition such as diabetes, hypertension, heart disease, or high blood cholesterol (Table 4). Among adults who had either hypertension or heart disease, a high percentage of adults who watched more than 2 hours of television were overweight compared with adults who watched less than 1 hour of television.

Significant differences were seen in the total energy intakes among the three television-viewing categories (Table 5). Adults who watched television for less than 1 hour per day had the lowest total energy intake. Conversely, adults who watched more than 2 hours of television had the highest energy intake; they consumed the highest amount of total fat, carbohydrate (including added sugars), and protein among the three television-viewing categories. Except for percentage of total calories obtained from added sugars, no differences were noted among the groups in the percentage of total calories obtained from other macronutrients. Adults who watched more than 2 hours of television per day consumed significantly less dietary fiber per 1000 kcal of total energy. However, this small difference is not of practical importance.

Adults who watched more than 2 hours of television per day also consumed high amounts of energy-rich snack-type foods, grain mixtures such as pizza, and regular soft drinks, which are sources of added sugars. Adults who watched more than 2 hours of television per day consumed more energy at supper and from snacks than adults who watched less than 1 hour of television per day.

No differences were noted between the mean energy and macronutrient intakes from the unadjusted models and adjusted (for age, sex, and other socioeconomic and demographic variables) regression models. Adding exercise status to the second regression models did not change the means.

## Discussion

A few broad conclusions emerged from the study. First, watching more than 2 hours of television per day was associated with having a high BMI and being overweight or obese in both men and women. In general, across all socioeconomic and demographic groups analyzed, with the exception of racial and ethnic groups, significantly higher percentages of adults who watched more than 2 hours of television per day were overweight compared with adults who watched less than 1 hour of television per day. Among racial and ethnic groups, television-viewing status varied significantly only between groups of white adults. Small sample sizes may explain the lack of differences among the other three racial and ethnic groups. Only a small percentage of African Americans, Hispanics, and "other races" watched less than 1 hour of television per day. The small sample sizes resulted in large standard errors. Also, some of the racial and ethnic categories used in this study were not homogeneous. For example, the Hispanic category included Mexican, Mexican American, Cuban, Puerto Rican, and other Hispanic; and the Other category included American Indians, Alaska Natives, Pacific Islanders, and Asians.

A high percentage of adults aged 50 or older watched more than 2 hours of television per day. Retirement may explain the more sedentary lifestyle, especially among adults aged 65 or older. Low household income, low level of education, and not being employed were associated with more adults watching more than 2 hours of television. More African American adults than white adults watched more than 2 hours of television. Having 4 years of college education or being employed was associated with a relatively low percentage of adults watching more than 2 hours of television per day. Similar findings were noted by Sidney et al (15). Using cross-sectional data from the Coronary Artery Risk Development in Young Adults (CARDIA) Study, they found that African Americans watched more hours of television per day than whites, and the number of hours of television viewed was inversely associated with education and income. Also, compared with those who watched less than 1 hour of television, the study found that those who watched more than 4 hours of television per day had a higher prevalence of obesity in all race and sex groups.

A significantly higher percentage of not-employed adults compared with employed adults watched more than 2 hours of television per day, indicating more available leisure time. However, among not-employed adults who were keeping house, a low percentage watched more than 2 hours of television per day, and a small percentage of them were overweight. Very likely, these adults had less leisure time available and were also physically active. Standing or walking around the home for 2 hours daily is associated with a 9% reduction in obesity in women (14).

Second, adults who had health conditions such as diabetes, hypertension, heart disease, or high blood cholesterol watched more than 2 hours of television per day, and a high percentage of them were also overweight. A small sample size (5.8% of the total population) may be the reason for not observing differences among adults with diabetes. Although the cross-sectional data provided by CSFII cannot be used to show cause and effect, the results show an association between health conditions, television viewing, and overweight status.

Many research studies have shown positive associations between prolonged television viewing and obesity and health conditions. Using data from a large prospective cohort study, Hu et al observed that prolonged television watching was positively associated with an increased risk of obesity and type 2 diabetes in men and women (13,14,25,26). From their study on women, they concluded that watching fewer than 10 hours of television per week and walking briskly for 30 minutes or more per day would prevent new cases of obesity in 30% of their cohort and prevent new cases of diabetes in 43% of the same cohort (14). Kronenberg et al (10) observed significant positive associations between television watching and obesity-related anthropometric measurements such as BMI, waist girth, waist–hip ratio, subscapular and triceps skinfold thickness, and atherosclerosis risk factors. Hubert et al (27) used a cross-sectional survey of Latino women aged 18 to 64 and found that watching television regularly was one of the significant factors associated with higher BMI (27). Fung et al, in a long-term study from 1986 to 1994 of 468 healthy male health professionals, found a positive association between television viewing and leptin levels and low-density lipoprotein cholesterol and an inverse association between television viewing and high-density lipoprotein cholesterol and apolipoprotein A1 (28).

Third, after adjusting for socioeconomic and demographic variables, adults who watched more than 2 hours of television per day consumed 137 calories more than adults who watched less than 1 hour of television per day. Assuming that the energy intakes and expenditures remain constant throughout the year for the adults who watch more than 2 hours of television per day and that they consume more calories than they expend, these 137 excess calories per day would translate into a gain of 14.3 pounds per year.

The CSFII collects information on the time of day each food or beverage is consumed, but it does not collect information on the time of day that television is watched. Therefore, it is not possible to provide direct evidence from this study that people surveyed ate while watching television or that watching television contributed to increased energy intakes. This is a limitation of the study. However, other studies have shown that people consume more food or energy while watching television. Gore et al reported that snacking (but not necessarily eating meals) while watching television was associated with increased total energy and energy from fat in women (20).

In this study, adults who watched more than 2 hours of television per day had significantly higher energy intakes at supper (46 more calories) and from snacks (61 more calories) and obtained a significantly higher percentage of energy from added sugars than those who watched less than 1 hour of television. Diets high in added sugars have been associated with poor nutritional quality (29). Adults who watched more than 2 hours of television per day also consumed more added sugar through regular soft drinks (81 more grams), the top source of added sugars in the United States (29,30) and ate more energy-rich snack-type foods such as cakes, cookies, corn chips, fried potatoes, and grain mixtures such as pizza. Snacking while watching television has been associated with increased daily energy intake and increased total fat calories (20).

The underlying causes of overweight and obesity are multidimensional. However, individuals from all socioeconomic backgrounds would benefit from reducing their television- or video-viewing time or time spent in similar sedentary activities. Instead of watching television, they may spend leisure time to increase their physical activity levels. People who are retired, disabled, or have health conditions may choose to do light- or moderate-intensity activities such as walking, stretching, and doing household chores. Others who are not limited by health conditions may engage in more vigorous or high-intensity physical activities.

Also, adults should be cognizant of the fact that eating while watching television could potentially increase their energy intakes substantially and may lead to positive energy balance. Therefore, adults should refrain from consuming energy-rich foods and beverages while watching television. Health interventions aimed at preventing or treating obesity should emphasize reducing time spent watching television or doing similar sedentary activities.

A lack of access to safe physical activity sites such as health spas, gyms, parks, walking trails, and swimming pools should not be a limiting factor for exercise or physical activity. A wide range of sports equipment such as treadmills, weight machines, and stationary bikes available in the market can be used in homes. Adults may also choose an exercise they may enjoy doing while watching television.

## Figures and Tables

**Table 1:** Mean Body Mass Index (BMI) and Percentage of Overweight or Obese Adults Aged 20 and Older, by Sex and Television-Viewing Status, Continuing Survey of Food Intakes by Individuals 1994–1996

**BMI Status**	**Men Mean (95% CI^a^)**			**Women Mean (95% CI^a^)**		

	**Watched <1 Hour Television per Day**	**Watched 1-2 Hours Television per Day**	**Watched >2 Hours Television per Day**	**Watched <1 Hour Television per Day**	**Watched 1-2 Hours Television per Day**	**Watched >2 Hours Television per Day**
BMI, kg/m^2^	25.4 (25.2-25.6)	26.1 (25.8-26.4)	26.8 (26.6-27.0)	24.7 (24.2-25.2)	25.0 (24.6-25.4)	26.4 (26.0-26.7)
Overweight or obese (BMI ≥25), %	50.8 (45.2-56.4)	60.4 (56.9-63.9)	62.3 (59.8-64.8)	39.2 (33.8-44.5)	39.6 (36.1-43.1)	52.8 (50.0-55.7)
Obese (BMI ≥30), %	10.3 (8.3-12.3)	14.2 (12.4-16.0)	19.6 (18.6-20.6)	15.7 (11.8-19.5)	14.1 (11.9-16.4)	22.2 (20.0-24.4)

a CI indicates confidence interval.

**Table 2:** Television-Viewing and Overweight Status Among Adults Aged 20 and Older, by Socioeconomic and Demographic Characteristics (N = 9157), Continuing Survey of Food Intakes by Individuals 1994–1996

**Characteristics**	**Weighted %**	**Watched >2 Hours Television per Day Weighted % (95% CI^a^)**	**Overweight or Obese AdultsWeighted % (95% CI^a^)**			

			**In Total Population**	**Watched <1 Hour Television per Day**	**Watched 1-2 Hours Television per Day**	**Watched >2 Hours Television per Day**

**Sex**						

Male	47.9	62.1 (59.6-64.6)	60.4 (58.2-62.6)	50.8 (45.2-56.4)	60.4 (56.9-63.9)	62.3 (59.8-64.8)
Female	52.1	55.9 (53.2-58.6)	47.0 (44.8-49.1)	39.2 (33.8-44.5)	39.6 (36.1-43.1)	52.8 (50.1-55.7)

**Age group, y**						

20-29	20.3	57.3 (53.7-60.9)	39.7 (36.6-42.9)	31.6 (24.9-39.0)	39.8 (33.4-46.1)	41.9 (37.5-46.3)
30-39	23.9	54.1 (50.6-57.6)	51.6 (47.0-56.4)	39.4 (31.6-47.1)	44.3 (39.4-49.2)	59.4 (53.3-65.5)
40-49	19.6	51.7 (48.5-54.9)	60.8 (57.9-63.8)	52.3 (44.3-60.2)	56.9 (51.9-61.9)	66.2 (62.5-69.8)
50-65	20.5	62.1 (59.8-64.4)	63.0 (60.6-65.4)	53.2 (45.6-60.8)	59.8 (56.1-64.5)	66.3 (63.6-68.9)
≥66	15.6	73.0 (69.8-76.2)	52.6 (39.6-55.8)	48.0 (39.6-56.4)	45.4 40.1-50.6)	54.9 (50.7-59.1)

**Education**						

High school or less	49.6	66.2 (63.8-68.6)	58.4 (56.4-60.3)	48.2 (42.3-54.0)	54.1 (49.5-58.6)	61.5 (58.7-64.3)
1-3 years college	23.4	56.5 (52.9-60.1)	50.6 (47.0-54.1)	44.7 (39.1-50.4)	48.7 (43.3-54.2)	53.1 (48.2-58.0)
4 years or more college	25.9	47.2 (44.2-50.2)	47.0 (44.2-49.8)	39.3 (32.9-45.6)	44.2 (43.3-54.1)	52.1 (48.1-58.0)

**Annual household income, % of federal poverty level**						

0-130	15.7	65.9 (61.4-70.4)	55.4 (51.6-59.1)	44.9 (37.6-52.2)	54.2 (48.1-60.3)	57.9 (53.5-62.2)
131-350	41.1	62.2 (59.2-63.2)	53.1 (50.8-55.4)	47.5 (41.8-53.2)	48.9 (44.5-53.3)	55.9 (53.4-58.3)
>350	43.2	53.1 (50.6-55.6)	53.2 (50.6-55.8)	40.9 (35.9-45.9)	48.4 (44.1-52.7)	59.7 (56.5-62.8)

**Race and ethnicity**						

White	76.0	58.5 (56.5-60.5)	52.3 (50.4-54.2)	40.7 (36.8-44.9)	49.5 (46.6-52.4)	56.5 (54.1-58.9)
African American	11.0	67.5 (63.1-71.9)	66.3 (62.8-69.8)	67.0 (55.8-78.2)	61.5 (52.4-70.5)	67.6 (63.6-71.7)
Hispanic	9.0	55.9 (53.8-58.0)	57.6 (52.3-62.8)	59.1 (50.4-67.8)	47.0 (31.8-62.2)	62.1 (58.1-66.0)
Other	4.0	47.8 (44.0-51.6)	31.3 (21.5-41.0)	26.6 (7.8-45.2)	28.2 (15.6-40.8)	35.3 (25.5-45.1)

**Urbanization**						

Metropolitan statistical area–central city	32.4	59.3 (55.3-63.3)	50.6 (47.0-54.2)	38.7 (31.8-45.6)	43.7 (39.0-48.3)	56.6 (52.6-60.5)
Metropolitan statistical area–suburban	46.2	58.8 (56.3-61.3)	45.9 (52.1-56.9)	45.9 (41.0-50.7)	51.8 (47.6-55.9)	57.9 (55.1-60.7)
Nonmetropolitan statistical area– rural	21.3	58.4 (53.2-63.6)	55.7 (53.2-58.2)	48.2 (38.8-57.7)	52.4 (45.8-59.0)	59.0 (53.6-64.4)

**Region**						

Northeast	20.1	59.5 (54.2-64.8)	53.9 (49.0-58.7)	35.9 (29.8-42.0)	50.7 (44.0-57.4)	59.6 (54.2-64.9)
Midwest	23.3	60.2 (56.2-64.2)	56.5 (53.8-59.2)	44.1 (36.4-51.8)	55.6 (51.2-60.0)	59.5 (55.9-63.1)
South	35.0	60.4 (57.2-63.6)	54.4 (51.4-57.5)	48.1 (41.3-54.8)	52.7 (48.9-56.6)	56.7 (53.3-60.2)
West	21.6	54.4 (49.1-59.6)	48.2 (43.5-52.9)	44.7 (36.7-52.7)	36.5 (31.3-41.7)	55.4 (49.1-61.6)

a CI indicates confidence interval.

**Table 3:** Television-Viewing and Overweight Status Among Adults Aged 20 or Older, by Employment Status, Continuing Survey of Food Intakes by Individuals 1994–1996

**Employment Status**	**Weighted %**	**Survey Population Weighted % (95% CI^a^)**			**Overweight or Obese Adults Weighted % (95% CI^a^)**		

		**Watched <1 Hour Television per Day**	**Watched 1-2 Hours Television per Day**	**Watched >2 Hours Television per Day**	**Watched <1 Hour Television per Day**	**Watched 1-2 Hours Television per Day**	**Watched >2 Hours Television per Day**
Employed full time	51.0	17.4 (16.0-18.8)	31.5 (29.3-33.7)	51.1 (48.7-53.5)	46.4 (42.8-50.0)	53.8 (50.4-57.2)	61.3 (57.8-60.0)
Employed part time	12.4	15.0 (12.0-18.0)	28.8 (25.6-32.0)	56.2 (51.8-60.6)	38.2 (33.2-43.2)	41.9 (34.7-49.0)	49.8 (43.3-56.4)
Not employed	36.6	11.7 (9.9-13.5)	18.7 (16.9-20.5)	69.6 (67.2-72.0)	42.6 (33.4-51.8)	42.5 (36.9-48.1)	57.0 (53.7-60.3)
Retired	17.0	7.7 (6.1-9.3)	18.1 (16.0-20.2)	74.2 (71.6-76.8)	49.2 (39.4-59.0)	45.8 (20.9-45.7)	57.7 (47.7-58.1)
House- keeper	8.5	14.8 (11.9-18.4)	21.5 (17.4-25.6)	63.7 (59.1-68.3)	36.0 (24.4-47.6)	33.3 (20.9-45.7)	52.9 (47.7-58.1)

a CI indicates confidence interval.

**Table 4:** Television-Viewing and Overweight Status Among Adults Aged 20 or Older, by Health Status, Continuing Survey of Food Intakes by Individuals 1994–1996

**Health Status**		**Weighted %**	**Watched >2 Hours Television Weighted % (95% CI^a^) **	**Overweight or Obese Adults Weighted % (95% CI^a^)**		

				**Watched <1 Hour Television per Day**	**Watched 1-2 Hours Television per Day**	**Watched >2 Hours Television per Day**
Has a doctor ever told you that you have diabetes?	Yes	5.8	70.8 (65.8-75.8)	67.9 (54.7-81.1)	68.5 (58.7-78.2)	76.5 (70.9-82.1)
	No	94.2	58.1 (55.9-60.3)	43.1 (39.2-47.0)	48.4 (45.5-51.2)	56.3 (54.1-58.5)
Has a doctor ever told you that you have hypertension?	Yes	20.8	69.0 (66.4-71.6)	61.3 (53.1-69.4)	68.7 (63.9-73.6)	72.9 (70.5-75.3)
	No	79.2	56.2 (54.0-58.4)	41.4 (37.1-45.6)	45.5 (42.4-48.5)	52.8 (50.2-55.4)
Has a doctor ever told you that you have heart disease?	Yes	7.8	70.4 (64.0-74.6)	47.6 (37.8-57.4)	49.8 (41.0-58.6)	62.9 (57.7-68.1)
	No	92.3	57.9 (55.7-60.1)	43.9 (40.1-47.7)	49.2 (46.4-52.0)	57.2 (55.0-59.3)
Has a doctor ever told you that you have high blood cholesterol?	Yes	14.6	67.4 (65.0-69.8)	62.7 (55.5-69.8)	60.5 (54.1-66.9)	68.8 (65.1-725.)
	No	85.4	57.4 (55.2-59.6)	42.2 (38.1-46.2)	47.6 (44.6-50.6)	55.4 (52.7-58.1)

a CI indicates confidence interval.

**Table 5:** Mean Nutrients and Food Intakes Among Adults Aged 20 or Older, by Television-Viewing Status, Continuing Survey of Food Intakes by Individuals 1994–1996

**Macronutrients and Food Groups**	**Unadjusted Mean (95% CI^a^)**			**Mean (95% CI^a^) Adjusted for Socioeconomic and Demographic Status^b^**		

	**Watched <1 Hour Television per Day**	**Watched 1-2 Hours Television per Day**	**Watched >2 Hours Television per Day**	**Watched <1 Hour Television per Day**	**Watched 1-2 Hours Television per Day**	**Watched >2 Hours Television per Day**
Total energy, kcal	1880 (1815-1944)	1966 (1934-1998)	2034 (1970-2098)	1896 (1838-1954)	1962 (1930-1993)	2033 (1983-2088)
Breakfast	331 (317-345)	327 (215-339)	345 (331-359)	342 (320-364)	332 (318-346)	340 (326-354)
Lunch	484 (460-508)	525 (499-551)	511 (491-531)	482 (458-506)	518 (494-542)	514 (496-532)
Supper	728 (698-758)	759 (731-787)	785 (759-811)	737 (715-759)	757 (735-779)	783 (761-805)
Snacks	327 (295-359)	347 (319-375)	387 (369-405)	329 (301-357)	340 (316-364)	390 (372-408)
Total fat, g	70.0 (67.4-72.5)	72.7 (70.7-74.7)	76.4 (74.4-78.4)	71.0 (69.0-73.0)	73.1 (71.1-75.1)	76.3 (74.3-78.3)
Saturated fat, g	23.2 (22.2-24.2)	24.0 (23.2-24.7)	25.9 (24.5-27.1)	23.4 (22.4-24.4)	23.9 (23.3-24.5)	25.7 (24.7-26.7)
Total carbohydrate, g	234 (225-243)	247 (242-252)	253 (246-260)	235 (229-241)	245 (242-248)	253 (247-259)
Added sugars^c^, g	66 (62-70)	72 (69-75)	79 (76-81)	65 (62-68)	71 (69-73)	79 (76-81)
Dietary fiber, g	16 (14.8-16.3)	16 (15.5-16.5)	16 (15.4-16.0)	16 (15.2-16.8)	16 (15.6-16.4)	16 (15.6-16.4)
Protein, g	73 (70.7-75.3)	76 (74.4-77.8)	80 (76.8-82.2)	74 (71.8-76.2)	76 (73.6-78.4)	79 (76.8-81.2)
Total fat kcal, %	33.1 (32.6-33.7)	32.8 (32.2-33.3)	33.2 (32.9-33.5)	33.3 (32.7-33.9)	32.9 (32.3-33.5)	33.1 (32.7-33.5)
Saturated fat kcal, %	10.9 (10.7-11.2)	10.8 (10.6-11.0)	11.1 (11.0-11.3)	11.0 (10.8-11.2)	10.8 (10.6-11.0)	11.1 (10.9-11.3)
Carbohydrate kcal, %	50.4 (49.5-51.2)	50.7 (50.2-51.2)	50.4 (50.0-50.8)	50.1 (49.7-50.4)	50.7 (50.3-51.1)	50.5 (50.1-50.9)
Added sugars kcal, %	13.8 (13.2-14.3)	14.3 (13.8-14.8)	15.1 (14.7-15.5)	13.6 (13.1-14.1)	14.3 (13.8-14.8)	15.2 (14.9-15.3)
Protein kcal, %	15.8 (15.5-16.1)	15.8 (15.6-16.1)	16.0 (15.8-16.1)	15.9 (15.7-16.1)	15.9 (15.7-16.1)	15.9 (15.7-16.1)
Dietary fiber per 1000 kcal, g	8.6 (8.4-8.8)	8.4 (8.2-8.6)	8.2 (8.0-8.3)	8.6 (8.4-8.8)	8.4 (8.2-8.6)	8.1 (8.0-8.2)
Regular soft drinks^d^, g	195 (175-215)	231 (210-252)	260 (238-282)	185 (165-205)	225 (206-244)	266 (247-285)
Energy-rich, snack-type foods^e^, g	90 (83-97)	101 (93-108)	105 (102-108)	89 (83-95)	93 (88-98)	100 (97-103)
Grain mixtures^f^, g	92 (84-100)	107 (101-113)	106 (101-111)	88 (80-96)	103 (97-106)	108 (102-114)

a CI indicates confidence interval.

b Means adjusted for age, sex, race, ethnicity, annual household income as percentage of poverty level, urbanization, and region.

c Added sugars include all sugars used as ingredients in processed and prepared foods, sugars added to foods at the table, and sugars eaten separately. Added sugars do not include sugars present naturally in foods such as lactose in milk and fructose in fruits.

d Regular soft drinks include all carbonated soft drinks except unsweetened and sugar-free types.

e Energy-rich snack-type foods include cakes, cookies, pastries, pies, sweet rolls, crackers, popcorn, pretzels, corn chips, and fried potatoes, including potato chips.

f Grain mixtures include food mixtures having a grain product as a main ingredient, such as pizza, burritos, tacos, egg rolls, quiche, spaghetti with sauce, rice, and pasta mixtures.
